# Immunoprecipitation methods impact the peptide repertoire in immunopeptidomics

**DOI:** 10.3389/fimmu.2023.1219720

**Published:** 2023-07-21

**Authors:** Marcel Wacker, Jens Bauer, Laura Wessling, Marissa Dubbelaar, Annika Nelde, Hans-Georg Rammensee, Juliane S. Walz

**Affiliations:** ^1^ Department of Peptide-based Immunotherapy, University and University Hospital Tübingen, Tübingen, Germany; ^2^ Institute for Cell Biology, Department of Immunology, University of Tübingen, Tübingen, Germany; ^3^ Cluster of Excellence iFIT (EXC2180) “Image-Guided and Functionally Instructed Tumor Therapies”, University of Tübingen, Tübingen, Germany; ^4^ Quantitative Biology Center (QBiC), University of Tübingen, Tübingen, Germany; ^5^ German Cancer Consortium (DKTK) and German Cancer Research Center (DKFZ), partner site Tübingen, Tübingen, Germany; ^6^ Clinical Collaboration Unit Translational Immunology, German Cancer Consortium (DKTK), Department of Internal Medicine, University Hospital Tübingen, Tübingen, Germany

**Keywords:** immunotherapies, HLA peptides, mass spectrometry, immunopeptidomics, immunoprecipitation, hydrophobicity, immunogenicity

## Abstract

**Introduction:**

Mass spectrometry-based immunopeptidomics is the only unbiased method to identify naturally presented HLA ligands, which is an indispensable prerequisite for characterizing novel tumor antigens for immunotherapeutic approaches. In recent years, improvements based on devices and methodology have been made to optimize sensitivity and throughput in immunopeptidomics. However, developments in ligand isolation, mass spectrometric analysis, and subsequent data processing can have a marked impact on the quality and quantity of immunopeptidomics data.

**Methods:**

In this work, we compared the immunopeptidome composition in terms of peptide yields, spectra quality, hydrophobicity, retention time, and immunogenicity of two established immunoprecipitation methods (column-based and 96-well-based) using cell lines as well as primary solid and hematological tumor samples.

**Results:**

Although, we identified comparable overall peptide yields, large proportions of method-exclusive peptides were detected with significantly higher hydrophobicity for the column-based method with potential implications for the identification of immunogenic tumor antigens. We showed that column preparation does not lose hydrophilic peptides in the hydrophilic washing step. In contrast, an additional 50% acetonitrile elution could partially regain lost hydrophobic peptides during 96-well preparation, suggesting a reduction of the bias towards the column-based method but not completely equalizing it.

**Discussion:**

Together, this work showed how different immunoprecipitation methods and their adaptions can impact the peptide repertoire of immunopeptidomic analysis and therefore the identification of potential tumor-associated antigens.

## 1 Introduction

Human leukocyte antigen (HLA)-presented peptides and their T cell recognition play a key role in the immune surveillance of malignant diseases (1, 2). Utilizing the respective tumor antigens to therapeutically induce anti-tumor T cell responses is the aim of various recent T cell-based immunotherapeutic approaches (3–6). Therefore, a critical step of these therapeutic approaches is correctly identifying suitable antigen targets recognized by the immune system and showing natural, high-frequent, and tumor-exclusive presentation on the tumor cell surface (7). Currently, the only methodology suitable for an unbiased identification and characterization of naturally presented HLA class I- and HLA class II-restricted peptides is mass spectrometry (MS)-based immunopeptidomics (8, 9). The three core steps for immunopeptidome analysis are first the co-immunoprecipitation (co-IP) of solubilized HLA-peptide complexes from cell or tissue lysates, followed by the isolation and purification of HLA-restricted peptides and the MS-based peptide sequencing by liquid chromatography-coupled tandem mass spectrometry (LC-MS/MS) (10–12). Finally, the data analysis of acquired peptide spectra is performed by database search tools (13–15) with an applied false discovery rate (FDR) to identify HLA-presented peptides (10, 11, 16). Of note, adjustments or changes in these steps, particularly the preparation method, can lead to methodological biases including altered qualitative and quantitative peptide yields (17–22), which might impact target peptide selection. Recently, a high-throughput co-IP method enabling the isolation of HLA ligands in a 96-well format was developed, which showed, additionally to increased throughput, high reproducibility and sensitivity (12). This method provides various alterations in lysis buffers, purification steps, and 96-well plate format compared to classical column-based methods (10), which could impact the quantitative and qualitative peptide yields. Recently, a modified protocol of a similar, C18-cartridge-based method, has been proposed which used higher percentages of acetonitrile (ACN) (19). Following the example of this publication, higher ACN elution concentrations were examined.

Thus, in this work, we compared cell line- and primary tumor sample-derived immunopeptidome data sets generated either with the column-based (10), the 96-well-based (12), or the modified 96-well-based (19) isolation method by comparing their associated immunopeptidome composition, in terms of peptide yields, spectra quality, retention time, predicted hydrophobicity, and predicted immunogenicity to investigate the influence of the isolation method on target peptide selection for the development of T cell-based immunotherapy approaches.

## 2 Materials and methods

### 2.1 Patient samples

Peripheral blood mononuclear cells (PBMCs) of a chronic lymphocytic leukemia (CLL) patient and solid tumor tissue of a renal cell carcinoma (RCC) patient were used for HLA ligand isolation by a column- and a 96-well-based preparation method and subsequent MS-based immunopeptidome analysis. Blood of the CLL patient was collected at the CCU Translational Immunology, Department of Internal Medicine, University Hospital Tübingen, Germany and PBMCs were isolated by density gradient centrifugation, snap frozen, and stored at -80°C until further use. Primary RCC tumor tissue was collected at the Department of Urology, University Hospital Tübingen, Germany, and stored at -80°C until further use. Informed consent was obtained according to the Declaration of Helsinki protocol. The study was performed according to the guidelines of the local ethics committees (406/2019B02, 424/2007B02). The Department of Hematology and Oncology, Tübingen, Germany and the Stefan Morsch Stiftung, Birkenfeld, Germany carried out HLA typing.

### 2.2 Cell line

The JY cell line (ECACC 94022533, batch 5070, HLA-A*02:01, -B*07:02, -C*07:02, -DRB1*04:04, -DRB1*13:01, -DQA1*01:03, -DQA1*03:01, -DQB1*03:02, -DQB1*06:03, -DPA1*01:03, -DPB1*02:01, -DPB1*04:01^1^) was cultivated in RPMI 1640 medium with 10% fetal calf serum (FCS) and 1% penicillin-streptomycin, harvested, washed 3x with phosphate-buffered saline (PBS), centrifuged down to pellets of 1x10^8^ cells and stored at -80°C until further us.

### 2.3 Immunopurification of HLA peptides

HLA immunopurifications were performed either as column-based (10) or 96-well-based (11, 12) preparation using the pan-HLA class I-specific monoclonal antibody (mAb) W6/32, the pan-HLA class II-specific mAb Tü-39, and the HLA-DR-specific mAb L243 (all produced in-house) to extract HLA ligands. All steps were performed at 4°C in a cold room.

#### 2.3.1 Column-based immunopurification of HLA peptides

For the cell lysis, 1.25 ml per 1x10^8^ cells or 7 ml per gram tissue of a 3-[(3-cholamidopropyl)dimethylammonio]-1-propanesulfonate (CHAPS)-based lysis buffer (1.2% (w/v) in PBS (pH 7.2); Panreac AppliChem, Darmstadt, Germany) were used. The masses of the tissue sample were determined and then immediately transferred to a petri dish, covered with lysis buffer, cut into thin slices using a scalpel, and homogenized in a homogenizer. Cell pellets or homogenized tissue samples were incubated in lysis buffer shaking for 1 hour, followed by ultra sonification (with at least 150 W of ultrasonic power, 50% pulse length, 2 minutes) and another subsequent incubation of 1 hour. Cell debris was cleared by centrifugation at maximum speed (3100 x g), followed by sterile filtration through a 5 µm filter. The column system consisted of two columns (Econo Column^®^ Chromatography Columns 0.5 cm × 5 cm BioRad, München, Germany) connected by tubing, where the upper column was used for the mAb W6/32 coupled to cyanobromide-activated sepharose beads (1 mg mAb was coupled to 40 mg beads suspended in 1 ml PBS (cyanobromide-activated sepharose 4B, Cytiva Sweden AB, Uppsala, Sweden)), and the lower column was used for the cyanobromide-activated sepharose beads coupled mAbs Tü-39 and L243 (mixture 1:1). The sample was circulated overnight through the column system containing 1 mg antibody per 1x10^8^ cells or per 0.83 gram tissue. Washing with PBS and double distilled water was performed, followed by transiently drying of the matrix. Four times acid elution were performed afterwards with transiently drying of the matrix in between the elution steps. In the first elution, 150 μl of 0.2% (v/v) trifluoro acetic acid (TFA) and 50 μl of 10% (v/v) TFA were used, followed by 150 μl of 0.2% (v/v) TFA in the last 3 repeats. The incubation time of acidic elution was 15 minutes for each of the four elution steps. All four eluates were combined and then filtered with 3 kDa and 10 kDa ultracentrifuge filters (Amicon Ultra 0.5 centrifugal filter unit 3 or 10 kDa, Merck Millipore, Billerica, USA) for HLA class I and HLA class II peptides, respectively. Filtrates were then frozen at -80°C and subsequently concentrated using a lyophilizer, followed by purification and desalting steps using a ZipTip C18 pipette tip (15 µm particle size, 200 Å pore size, 0.6 µl volume, Merck-Milipore, Darmstadt, Germany). After binding of peptides to the C18 ZipTip, the tip was washed in 0.1% (v/v) TFA, and the peptides were subsequently eluted in 32% (v/v) ACN in 0.2% (v/v) TFA. The 0.1% (v/v) TFA washing solution (termed desalting) was also investigated further to determine a potential loss of peptides during washing. The desalting and final sample volumes were reduced with vacuum centrifugation and filled up to a volume of 25 µl with 1% (v/v) ACN in 0.05% (v/v) TFA and subsequently analyzed by LC-MS/MS.

#### 2.3.2 Desalting step

The lyophilized filtrates were desalted with a ZipTip C18 pipette tip during the column-based preparation method. Before the peptides were eluted in 32% (v/v) ACN in 0.2% (v/v) TFA, a washing step was performed in 0.1% (v/v) TFA. The liquid of the washing solution was lyophilized and filled up to a volume of 25 µl with 1% (v/v) ACN in 0.05% (v/v) TFA and separately analyzed by LC-MS/MS.

#### 2.3.3 96-well-based immunopurification of HLA peptides

The 96-well-based preparation lysis buffer consisted of sodium deoxycholate (0.25% (w/v); Sigma-Aldrich, Steinheim am Albuch, Germany) and octyl-beta-D glucopyranoside (1% (w/v); Sigma-Aldrich) in PBS (pH 7.2). 1 ml per 10^8^ cells or 9 ml per gram tissue of lysis buffer were used. The masses of the tissue sample were determined and immediately transferred to a petri dish, covered with lysis buffer, cut into thin slices using a scalpel, and homogenized in a homogenizer. Cell pellets or homogenized tissue samples were incubated in lysis buffer shaking for 1 hour, followed by ultra sonification (with at least 150 W of ultrasonic power, 50% pulse length, 2 minutes) and another subsequent incubation of 1 hour. Cell debris was cleared by centrifugation at maximum speed (3100 x g), followed by sterile filtration through a 5 µm filter. An upper 96-well plate (Polypropylene 96-well filter-micro plates, Agilent Technologies, Santa Clara, USA, 3 μm fiberglass, 25 μm polyethylene membrane) was filled with the mAb W6/32 crosslinked to protein A sepharose beads (1 mg mAb was coupled to 200 µl beads (Protein A-Sepharose 4B, Invitrogen Rockford, IL, USA)), and a lower 96-well plate with the mAbs Tü-29 and L243 (1:1 mixture) crosslinked to protein A sepharose beads. For the immunoprecipitation-step, the lysates were loaded on both plates by gravity containing 1 mg antibody per 1x10^8^ cells or per 0.83 gram tissue. Washing of samples followed, where several washing steps with different concentrations of Tris-HCl/NaCl (4x 150 mM sodium chloride (NaCl) in 20 mM Tris-HCl pH 8; 4x 400 mM NaCl in 20 mM Tris-HCl pH 8; 4x 150 mM NaCl in 20 mM Tris-HCl pH 8; 2x 20 mM Tris-HCl pH 8) were done. Acidic elution was performed directly with 500 μl 1% TFA (v/v) onto C18 plates (Sep-Pak^®^ tC18 100 mg, 37-55 µm particle size, 125 Å pore size, 96-Well-plates, Waters, Milford MA, USA), followed by hydrophobic elution with 500 μl 28% (v/v) or 32% (v/v) ACN in 0.1% (v/v) TFA for HLA class I or HLA class II peptides into collection plates, respectively. All eluates were frozen at -80°C, concentrated in a lyophilizer, and filled up to a volume of 25 µl with 1% (v/v) ACN in 0.05% (v/v) TFA and subsequently analyzed by LC-MS/MS.

#### 2.3.4 Adapted 96-well-based immunopurification of HLA peptides with 50% ACN elution step

For the adapted 96-well-based immunopurification of HLA peptides with 50% ACN elution step, another elution from the same C18 plates was performed after the hydrophobic elution of C18-bound peptides with 500 μl 28% (v/v) or 32% (v/v) ACN in 0.1% (v/v) TFA for HLA class I or HLA class II peptides into collection plates. This additional elution was performed with 50% (v/v) ACN in 0.1% (v/v) TFA in a new collection plate for HLA class I or HLA class II, respectively. The sample was frozen at -80°C, concentrated in a lyophilizer, and filled up to a volume of 25 µl with 1% (v/v) ACN in 0.05% (v/v) TFA and subsequently analyzed by LC-MS/MS.

### 2.4 Mass spectrometry-based analysis

Reversed-phase liquid chromatography (nanoUHPLC, UltiMate 3000 RSLCnano, Thermo Fisher, Waltham, Massachusetts, USA) was used for peptide separation, followed by an on-line coupled Q Exactive HF mass spectrometer (Thermo Fisher). Samples were analyzed in three technical replicates, where 5 µl with shares of 20% were injected onto a 75 µm x 2 cm trapping column (Thermo Fisher, Waltham, Massachusetts, USA) at 4 µl/min for 5.75 min with 1% (v/v) ACN in 0.05% (v/v) TFA as loading buffer followed by peptide separation at 50°C and a flow rate of 300 nL/min on a 50 µm x 25 cm separation column with 2 µm particle size (PepMap C18, Thermo Fisher) applying a gradient ranging from 2.4% to 32.0% of ACN over 90 min. Ionization of eluting peptides was conducted by a nanospray source and analysis occurred in the on-line coupled mass spectrometer by implementing a top 35 HCD (Higher-energy C-trap dissociation) method generating fragment spectra with a resolution of 30,000, a mass range limited to 400-650 m/z for HLA class I peptides and 400-1000 m/z for HLA class II peptides, and positive charge states 2–3 for HLA class I and 2–5 for HLA class II were selected for fragmentation.

### 2.5 Data processing

Data processing was performed as described previously (10). Integrating database search results of the SequestHT search engine [University of Washington (14)] against the human proteome (Swiss-Prot database, 20,279 reviewed protein sequences, September 27th, 2013) was performed by the Proteome Discoverer (v1.4, Thermo Fisher), using a precursor mass tolerance of 5 ppm, fragment mass tolerance of 0.02 Da, and allowing oxidized methionine as a dynamic modification. HLA class I and HLA class II peptides for the JY cell line, and primary tumor samples of CLL and RCC patients were co-processed, respectively. 1 co-processed dataset was composed of 1 biological, 1 technical preparation and 3 technical MS replicates, respectively. The false discovery rate (FDR, estimated by the Percolator algorithm 2.04 (23)) was limited to 5% for HLA class I and 1% for HLA class II. Identified peptides were filtered for 8-12 or 12-21 amino acids length for HLA class I or HLA class II. HLA class I binder analysis was performed using SYFPEITHI 1.0 (24) (% of max. score ≥ 60) and NetMHCpan 4.1 (25) (percentile rank ≤ 2). Either one or both of the predictions had to meet the binder criteria for the ligand to be included into the HLA class I data set. HLA class II binder analysis was performed using NetMHCIIpan 4.1^2^ (26) where the predictions had to meet the binder criteria of a percentile rank ≤ 5.

### 2.6 Software and statistical analysis

All figures and statistical analyses were generated using GraphPad Prism 9.4.0 (GraphPad Software). P values of < 0.05 were considered statistically significant. Overlap analyses were performed with InteraciVenn (27). Grand average of hydropathy (GRAVY) scores were calculated with a GRAVY calculator^3^ (28).

To analyze previously described tumor-associated antigens, datasets from CLL- (29–31) and RCC-related publications (32–34) were filtered for the HLA class I types of the respective sample. All HLA class II peptides within the length filters of 12-21 amino acids of the mentioned publications were used for the analysis.

The Immune Epitope Database (IEDB) (35) was filtered for linear peptides, MHC ligand (positive) in Homo sapiens (human) (ID:9606) with an MHC restriction for either HLA class I or HLA class II. Human was selected as the host, and either cancer (ID : DOID:162) or healthy (ID : ONTIE:0003423) was used as a filter for disease. Furthermore, peptides > 12 amino acids or < 8 amino acids were excluded for HLA class I as well as peptides > 21 amino acids or < 12 amino acids for HLA class II.

For the predicted immunogenicity calculation, column- or 96-well-based method-exclusive 9-mer peptides were analyzed with the “Class I immunogenicity” prediction tool on the IEDB^4^.

### 2.7 Data availability

The mass spectrometry proteomics data have been deposited to the ProteomeXchange Consortium *via* the PRIDE (36) partner repository with the dataset identifier PXD041804.

## 3 Results

### 3.1 Column-based and 96-well-based immunoprecipitation methods show a large proportion of method-exclusive peptides

**Figure 1:**
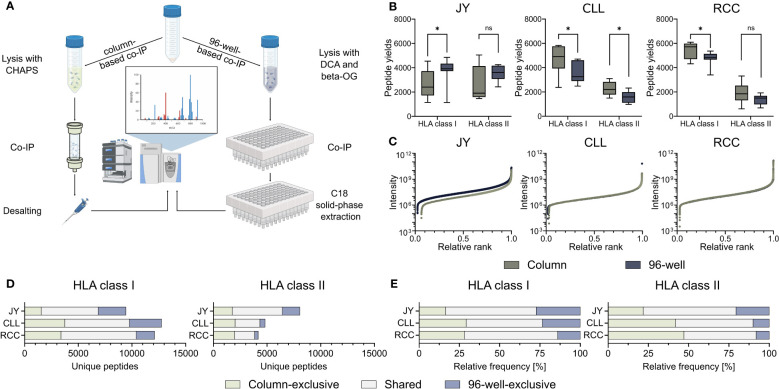
Comparison of 96-well- and column-based immunoprecipitation methods. **(A)** Schematic overview of the column- (left) and 96-well-based (right) co-immunoprecipitation (co-IP) methods. For the column-based method, a 3-[(3-cholamidopropyl)dimethylammonio]-1-propanesulfonate (CHAPS) lysis buffer was used, co-IP was performed in columns with cyclic samples overnight, and the eluted samples were finally desalted by a C18 pipet tip filter (ZipTip^®^). For the 96-well-based method, samples were lysed with a deoxycholic acid (DCA) and octyl-β-D-glucopyranoside (β-OG) buffer, and co-IP was performed in a 96-well system. The eluted samples were bound by C18 columns in a 96-well plate, and the peptides were eluted with acetonitrile (ACN). Samples from both preparation methods were measured using the same mass spectrometer (MS) device and method. Created with BioRender.com. **(B)** HLA class I and HLA class II peptide yields for the JY cell line (left panel), and primary tumor samples of a chronic lymphocytic leukemia (CLL, middle panel) and a renal cell carcinoma (RCC, right panel) patient (n = 9 co-processed datasets for each specimen and HLA class (**Supplementary Figure 1**, **Supplementary Table 1**). Green depicts all column-based peptides, light green column-based exclusive peptides; gray-blue depicts all 96-well peptides; light gray-blue depicts 96-well exclusive peptides. Boxes represent the median and 25^th^ to 75^th^ percentiles, whiskers are minimum to maximum. Unpaired t-tests, *p<0.05, ns not significant. **(C)** Relative ranked intensities of MS-acquired data of JY, CLL and RCC derived peptides from the combined HLA class I immunopeptidomes of all samples (n = 9), respectively. **(D, E)** Unique **(D)** absolute and **(E)** relative HLA class I (left panel) and HLA class II (right panel) peptide yields of JY, CLL and RCC identified by the column- and/or the 96-well-based method.

To investigate the influence on identified HLA-restricted peptides of the column- and 96-well-based co-IP methods, we performed immunopeptidome analysis from biological triplicates of the JY cell line as well as from a primary CLL and RCC sample, respectively. Therefore, immunoprecipitation and MS analyses were performed in technical triplicates, resulting in 27 HLA class I and 27 HLA class II single MS measurements per specimen (**Figure 1A**, **Supplementary Figure 1**, **Supplementary Table 1**). HLA class I peptide yields, in terms of unique identified peptides were significantly higher with the 96-well preparation for the JY sample (median column 2406, 96-well 3918). In contrast, the column preparation revealed significantly higher HLA class I peptide yields for the CLL (median column 4916, 96-well 3259) and RCC (median column 5719, 96-well 4817) specimens (**Figure 1B**). For HLA class II peptide yields, only for the CLL sample (median column 1964, 96-well 1418), a significantly higher peptide yield was detected with the column preparation. In contrast, for JY (median column 1696, 96-well 3227) and RCC (median column 1651, 96-well 1335), no significant difference was observed between the two methods (**Figure 1B**). The spectra quality, intensity distribution of the identified HLA class I and HLA class II peptides and reproducibility were similar between the two investigated methods in all three specimens (**Figure 1C**, **Supplementary Figure 2A–C**, **Supplementary Tables 2**, **3**). Only minor differences between XCorr values were detected, with no clear trend towards a method. Focusing on the reproducibility of the column-based and the 96-well-based method, a mean of 58.3% and 61.8% of the identified ligands were represented in at least three of the nine technical replicates, respectively. Of note, comparing the identified peptide sequences revealed a high proportion of method-exclusive peptides (**Figures 1D, E**). For HLA class I, 1553 (16.5%), 3735 (29.3%), and 3389 (28.0%) column-exclusive and 2575 (27.3%), 3003 (23.5%), and 1695 (14.0%) 96-well-exclusive peptides were detected of JY, CLL, and RCC samples, respectively. For HLA class II, there were 1759 (21.8%), 2015 (41.8%), 1969 (47.2%) column-exclusive, and 1649 (20.5%), 480 (10.0%), and 331 (7.9%) 96-well exclusive peptides of JY, CLL, and RCC samples, respectively (**Figures 1D, E**). In total, up to 47.2% of the identified peptides were method-exclusive.

### 3.2 Peptides isolated with column-based immunoprecipitation showed overall higher hydrophobicity scores

**Figure 2:**
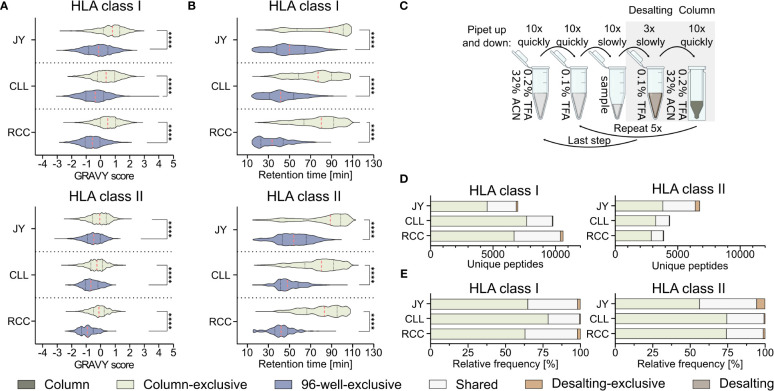
Influence of the immunoprecipitation method on the hydrophobicity of isolated peptides. **(A, B)** Violin plots of **(A)** grand average of hydropathy (GRAVY) scores and **(B)** retention times of column and 96-well preparation method-exclusive HLA class I (upper panel) and HLA class II (lower panel) peptides of JY, CLL, and RCC. Red dashed lines show the median, black dotted lines the 25^th^ and 75^th^ percentiles. ****p<0.0001. **(A)** Unpaired t-tests, **(B)** Mann-Whitney tests. **(C)** Schematic illustration of the desalting step during the column-based method, conducted with a C18 pipet filter tip (ZipTip^®^). Before hydrophobic elution of peptides occurred in 32% ACN (green), the filter tip was washed in 0.1% TFA, (brown, desalting). Immunopeptidome analyses were performed from the two gray underlaid conditions (column-based and desalting). Created with BioRender.com. **(D, E)** Unique peptide identification and frequency bar plots of column-exclusive (light green) or desalting wash step exclusive (light brown) peptides and shared peptides (light gray). Absolute **(D)** and relative **(E)** frequency of unique HLA class I (left panel) and HLA class II (right panel) peptides. Peptides unique to the column-based method are shown in light-green, peptides unique to the 96-well method are in gray-blue, peptides unique to the desalting step are in light brown and peptides found by the column method and the desalting wash step in light gray.

Further analysis of the method-exclusive peptides revealed a significant increase of peptide sequences with higher predicted hydrophobicity (**Figure 2A**) and, consequently also a shifted measured retention time (**Figure 2B**), for the column preparation compared to the 96-well preparation (**Supplementary Table 4**). The median of the calculated grand average of hydropathy (GRAVY) scores (28) of the column-exclusive HLA class I peptides was 0.8, 0.4, and 0.5 of JY, CLL, and RCC samples, and -0.1, -0.4 and -0.6, for the 96-well-exclusive peptides (**Figure 2A**). For HLA class II, the median GRAVY scores of the column-exclusive peptides were -0.1, -0.3 and -0.1 and -0.5, -0.7 and -0.9 for the 96-well-exclusive peptides of JY, CLL and RCC samples, respectively (**Figure 2A**). In line, the measured retention times of the column-exclusive peptides were significantly shifted towards later retention times compared to 96-well-exclusive peptides (**Figure 2B**). These effects were not only observed for method-exclusive but similarly for the entirety of identified peptides with significant differences in GRAVY scores and retention times for both HLA class I and HLA class II with significantly more hydrophobic peptides obtained with the column preparation (**Supplementary Figures 3A, B**, **Supplementary Tables 2**, **3**).

Since it is unclear from these data, whether this shift is caused by the absence of hydrophilic peptides in the column-based or the absence of hydrophobic peptides in the 96-well-method, we further investigated the most hydrophilic step of the column-based method and the most hydrophobic step of the 96-well-based process. The most hydrophilic step in the column-based method is the washing step in 0.1% (v/v) TFA during the ZipTip C18-based desalting step (referred to as desalting) (**Figure 2C**). Only 136 (1.9%), 55 (0.6%), and 213 (2.0%) HLA class I and 370 (5.5%), 25 (0.6%), and 47 (1.2%) HLA class II peptides were exclusively detected in the desalting step of JY, CLL, and RCC samples, respectively. The majority of the peptides identified in the desalting solution were overlapping with the peptides also detected with the column preparation only (**Figures 2D, E**). The GRAVY scores of these desalting-exclusive peptides were in general lower, thus more hydrophilic (**Supplementary Figure 3C**). In line, desalting-exclusive peptides elute significantly earlier (**Supplementary Figure 3D**). Based on the low number of desalting-exclusive peptides, the desalting step of the column-based method did not lead to the loss of hydrophilic peptides and was not responsible for the hydrophobicity shift between the column- and 96-well-based co-IP methods.

### 3.3 The loss of hydrophobic peptides can partially be restored with higher acetonitrile percentage

**Figure 3:**
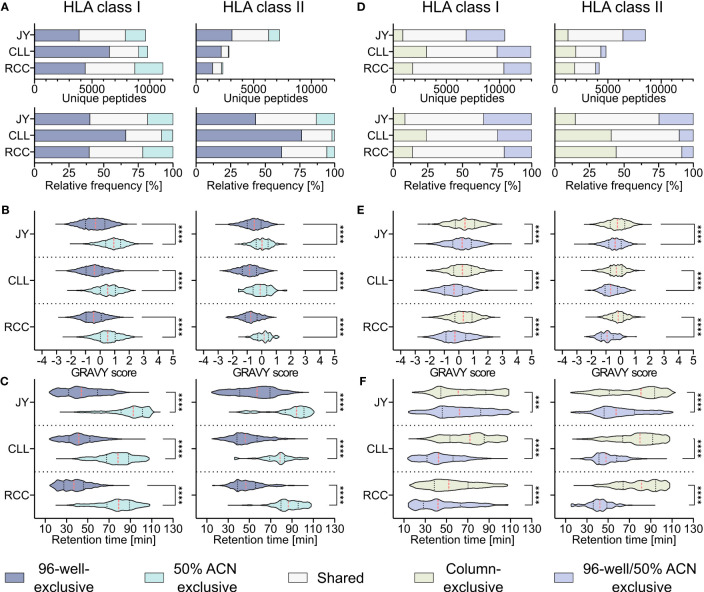
Effect of a second elution step with 50% acetonitrile (ACN) in the 96-well method. **(A)** Absolute (upper panels) and relative (lower panels) HLA class I (left panels) and HLA class II (right panels) peptide yields of JY, CLL and RCC samples identified by the 96-well-based method and/or the additional elution step with 50% ACN from the same 96-well plate. **(B, C)** Violin plots of **(B)** GRAVY scores and **(C)** retention times of 96-well-exclusive (gray-blue), 50% ACN-exclusive (turquoise) HLA class I (left panel) and HLA class II (right panel) peptides of JY, CLL, and RCC. Red dashed lines show the median, black dotted lines the 25^th^ and 75^th^ percentiles. ****p<0.0001. **(B)** Unpaired t-tests, **(C)** Mann-Whitney tests. **(D)** Absolute (upper panels) and relative (lower panels) HLA class I (left panels) and HLA class II (right panels) peptides and percentage of JY, CLL and RCC peptide yields identified by the column-based method (light green), or the 96-well method combined with the subsequent elution step with 50% ACN from the same 96-well plate (light gray-blue) or both (light gray). **(E, F)** Violin plots of **(E)** GRAVY scores and **(F)** retention times of column-based method-exclusive (light green), combination of 96-well method and 50% ACN exclusive (light gray-blue) HLA class I (left panel) and HLA class II (right panel) peptides of JY, CLL, and RCC. Red dashed lines show the median and black dotted lines show the 25^th^ and 75^th^ percentiles. ***p<0.001, ****p<0.0001. **(E)** Unpaired t-tests, **(F)** Mann-Whitney tests.

To investigate whether the hydrophobicity shift was due to the loss of hydrophobic peptides with the 96-well-based co-IP method, a modified protocol introducing a second elution step of the same C18 plates after the 28/32% ACN elution with 50% ACN was performed as described before (19). This second elution step resulted in up to 26% other HLA class I (JY 18% (1760/9640), CLL 8% (794/9819), RCC 21% (2426/11131)) and HLA class II (JY 13% (956/7254), CLL 2% (51/2853), RCC 5% (127/2332)) peptide identifications compared to the unmodified 96-well method (**Figure 3A**) and enabled the additional isolation of highly hydrophobic peptides (**Figures 3B, C**). GRAVY scores referring to the hydrophobicity of the 50% ACN-exclusive peptides, were significantly higher with medians of 0.9, 0.5 and 0.5 for HLA class I peptides and 0.0, -0.1 and 0.2 for HLA class II of JY, CLL and RCC samples, compared to the conventional 96-well preparation-exclusive peptides with medians of -0.3, -0.4 and -0.5 for HLA class I and -0.5, -0.8 and -0.8 for HLA class II (**Figure 3B**), respectively. In line, the median of the retention times of 50% ACN-exclusive peptides shifted by up to 49 minutes for HLA class I and up to 40 minutes for HLA class II towards later elution times compared to 96-well-exclusive peptides (**Figure 3C**). The same effects were not only observed for method-exclusive but also the entirety of identified peptides, showing significantly increased GRAVY scores and retention times for the 50% ACN elution compared to the 96-well preparation for both HLA class I and HLA class II (**Supplementary Figure 4**).

To examine whether a subsequent elution step with 50% ACN in the 96-well method could rescue the missing hydrophobic peptides compared to the column-based method, the 96-well preparation peptides and the peptides found by eluting a second time with 50% ACN (combination further called 96-well 50% ACN) were compared with the column preparation. However, up to 24% and 44% of the identified HLA class I (JY 9% (895/10475), CLL 24% (3108/12931), RCC 14% (1817/12948)) and HLA class II (JY 15% (1255/8509), CLL 41% (1968/4821), RCC 44% (1852/4184)) peptides identified in the column-based method remain exclusive even when the 96-well method is supplemented with the 50% ACN elution step (**Figure 3D**). Additionally, a significant difference in the hydrophobicity regarding GRAVY score and retention times was still observed for the method-exclusive peptides, albeit reduced compared to the 96-well method without the additional 50% ACN elution (**Figures 3E, F**) emphasizing the benefit of this method adaption. A global analysis of the researched methods and method adaptions (column, desalting, 96-well, 50% ACN) showed that peptide yields are not influenced by hydrophobic or hydrophilic binding motifs of corresponding HLA allotypes, thus do not influence peptide yields. However, allotypes with more hydrophobic binding motifs tend to present more hydrophobic peptides and vice versa (**Supplementary Figures 5A–C**).

### 3.4 Different immunoprecipitation methods show a bias in the identification of tumor-associated antigens

**Figure 4:**
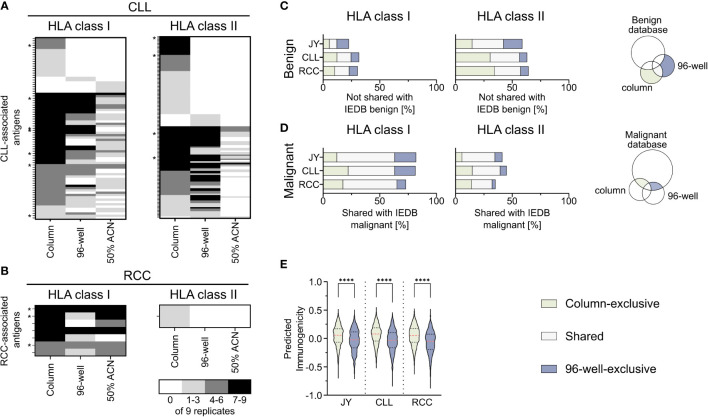
Comparison of identified peptides with published databases. **(A, B)** Heat map depicting previously described HLA class I (left panel) and HLA class II (right panel) tumor-associated antigens (TAAs), which were reidentified at least once in the immunopeptidomes of the analyzed **(A)** chronic lymphocytic leukemia (CLL, n = 9) and **(B)** renal cell carcinoma (RCC, n = 9) samples. The gray color intensity indicates the frequency of the respective peptide in the immunopeptidome replicates. * indicates a tumor-associated peptide with proven immunogenicity in the respective publication. **(C, D)** Relative overlaps of the column- or 96-well-exclusive HLA class I (left panel) and HLA class II (middle panel) peptides with **(C)** the benign and **(D)** the cancer-associated IEDB database. Percentages refer to the combined total of the unique column and 96-well peptides. A schematic Venn diagram (right panel) indicates which peptides are depicted in the bar plots. **(E)** Violin plot of predicted immunogenicity scores for 9-mers peptides within the HLA class I column- (light green) and 96-well-exclusive (light gray-blue) peptides. Red dashed lines show the median, black dotted lines the 25^th^ and 75^th^ percentiles. ****p<0.0001. Unpaired t-tests, ****p<0.0001.

To further investigate the impact of the used co-IP methods on the immunopeptidome-based identification of tumor-associated antigens, a comparative analysis of previously described CLL- (29–31) and RCC-associated TAAs (32–34), the IEDB and the here identified peptides was performed (**Supplementary Table 5**). Of the HLA-matched previously described CLL-associated HLA class I TAAs, 53% (79/149) could be reidentified in our analysis with at least one of the used methods (column, 96-well, or 50% ACN method), while 7 of the reidentified peptides were shown to be immunogenic in previous publications. Interestingly, 22% (17/79) of the peptides were exclusively identified with the column-based preparation method, whereas only 3% (2/79) and 6% (5/79) were identified solely with the 96-well-preparation method and the 50% ACN elution step, respectively (**Figure 4A**). Of the previously described CLL-associated HLA class II TAAs, 21% (135/643) could be reidentified with at least one method, and the same bias could be observed with 43% (58/135), 7% (9/135) and 0% (0/135) identified exclusively with the column-based method, the 96- well-based method and the 50% ACN elution step (**Figure 4A**). 4 of the re-identified peptides were immunogenic in previous publications. Of the HLA-matched previously described RCC-associated HLA class I and HLA class II TAAs, 70% (7/10) and 10% (1/10) could be reidentified in the RCC sample, respectively. None of the HLA class I peptides could be identified exclusively with one method and 3 of the reidentified peptides were immunogenic in previous publications. However, one peptide was reidentified with the column-based method and with 50% ACN elution but not with the 96-well-based method. The one HLA class II peptide could be identified exclusively with the column-based method (**Figure 4B**).

Furthermore, to examine the characteristics of the column-based or 96-well-based method in terms of the identification of TAAs, we compared the here identified peptides with the benign- and tumor-associated peptides described in the IEDB. Comparing the column and 96-well method-derived peptides with the described benign peptides showed a similar percentage of method-exclusive HLA class I peptides not found within the benign IEDB dataset (**Figure 4C**). Similarly, comparing the column and 96-well method-derived peptides with the malignant IEDB showed a similar percentage of method-exclusive HLA class I peptides also found in the malignant IEDB and therefore, similar ratios of method-exclusive TAAs (**Figure 4D**, **Supplementary Table 6**). For HLA class II, the ratio of the method-exclusive peptides acted similarly to HLA class I peptide rations. However, column-based method-exclusive peptides percentages were larger than those with the 96-well preparation for CLL and RCC. These distributions resemble the original relative distribution (**Figure 1E**). When these points are taken together, each method shows an equal potential to expand the IEDB database and discover tumor-associated antigens.

To further evaluate the impact of the co-IP methods on the immunogenicity of immunopeptidome-identified peptides, we predicted the immunogenicity of the column- or 96-well method-exclusive 9-mer peptides (**Figure 4E**, **Supplementary Table 7**). For all specimens, the median predicted immunogenicity of column preparation exclusive peptides was significantly higher compared to 96-well preparation peptides.

## 4 Discussion

Mass spectrometry-based immunopeptidomics is the only unbiased method to identify naturally presented HLA ligands (8, 9), which is an indispensable prerequisite for the characterization of novel tumor antigens for immunotherapeutic approaches (3–6, 37, 38). Immense improvements based on devices and methodology have been made in recent years to optimize sensitivity and sample throughput (12, 21, 39, 40). However, novel isolation methods, mass spectrometric devices and data processing pipelines and tools can have a marked impact on the quality and quantity of immunopeptidomics data and identified peptides (19, 20, 22, 41).

In this work, we performed a head-to-head comparison of two established immunoprecipitation methods that differ significantly in their purification steps to understand the bias that might be introduced by using these different methods (10, 12). Whereas peptide yields, spectra quality and reproducibility were comparable, a large proportion of method-exclusive peptides were identified with significant differences in their hydrophobicity, which might have potential implications for the identification of immunogenic tumor antigens.

Regarding peptide yields, no general trend towards higher yields of one of the methods was observed between the column-based and the 96-well-based method across all samples. The acquired variation in peptide yields might be due to the usage of different detergents in the lysis buffers of both methods (22). Although no tendency in terms of peptide yields was detected, with the 96-well method showing a trend of higher reproducibility, tremendous fractions of the identified peptides were exclusively detected in one of the methods. This was based on an increased number of hydrophobic peptides identified with the column method, which were not identified by the 96-well-based method. These findings align with previous studies that have reported alterations in peptide composition and/or hydrophobicity with different HLA-peptide isolation methods. Differences in salt concentrations during washing steps, lysis buffers, elution methods, or the use of different C18 based purification methods were described as main sources of method induced biases (19, 21, 22, 42). Specifically, the latter two can have a particular impact on hydrophobicity, as the use of different ACN percentages or different C18 materials have different properties to elute or bind hydrophobic peptides. Interestingly, method-specific peptide yields were not impacted by specific HLA allotypes, despite the allotype-specific hydrophobicity of the corresponding anchor amino acids, however the GRAVY score distribution showed the same method specific bias.

Since the number of open-access immunopeptidomic data is increasingly growing, these alterations in identified peptide repertoire based on different immunoprecipitation methods can have a marked impact on our knowledge about the immunopeptidome. In particular, selecting tumor-exclusive HLA peptides based on the subtraction of benign tissue immunopeptidome repositories could be biased using datasets generated with different immunoprecipitation methods. This becomes even more apparent within a specific search of previously published TAAs (29–34) identified with the column method within our dataset. These TAAs were preferentially detected in samples examined with the column method and were underrepresented in 96-well examined samples. Moreover, as we and others have shown, hydrophobic peptides tend to be more immunogenic (43, 44), and the immunoprecipitation method might also significantly impact the identification of T cell epitopes in individual tumor samples.

With an in-depth analysis of the purification steps, we could show that the shift in hydrophobicity is not caused by a loss of hydrophilic peptides during the hydrophilic washing step of the column preparation but generated by the loss of hydrophobic peptides during 96-well preparation, which can be partially overcome by increased ACN fractions for peptide elution. This underlines the positive effect of an additional elution step with increased ACN percentages, which is in line with previous reports showing that higher ACN proportion can increase peptide yields as well as the hydrophobicity and thus immunogenicity of peptide identifications (19, 21, 22). Nonetheless, we could show that column-based method-exclusive peptides still had significantly higher hydrophobicity, suggesting that 50% ACN elution in the 96-well method could reduce the bias towards the column-based method but not completely equalize it, further underlining the importance of knowing about method-specific biases.

Together, this work showed how different immunoprecipitation methods and their adaptions can impact the immunopeptidome composition in terms of hydrophobicity, retention time and immunogenicity and thus the identification of potential TAA.

## Data availability statement

The datasets presented in this study can be found in online repositories. The names of the repository/repositories and accession number(s) can be found below: https://www.ebi.ac.uk/pride/archive/, PXD041804.

## Ethics statement

The studies involving human participants were reviewed and approved by the Ethics Committee of the faculty of medicine of the University of Tübingen. The patients/participants provided their written informed consent to participate in this study.

## Author contributions

MW, JB, AN, and JSW conceptualized this study. MW, JB, and LW performed immunopeptidome experiments. MD performed bioinformatic analysis. AN, H-GR, and JSW supervised this study. All authors contributed to the article and approved the submitted version.

## Footnotes

1
https://www.ebi.ac.uk/ipd/imgt/hla/cells/cell/?cellid=10882, 05.06.2023

2
https://services.healthtech.dtu.dk/services/NetMHCIIpan-4.1/

3
http://www.gravy-calculator.de/

4
http://tools.iedb.org/immunogenicity/

## References

[1] Ryschich E Notzel T Hinz U Autschbach F Ferguson J Simon I. Control of T-cell-mediated immune response by HLA class I in human pancreatic carcinoma. Clin Cancer Res (2005) 11(2 Pt 1):498–504. doi: 10.1158/1078-0432.498.11.2 15701833

[2] Gao Q Liang WW Foltz SM Mutharasu G Jayasinghe RG Cao S. Driver fusions and their implications in the development and treatment of human cancers. Cell Rep (2018) 23(1):227–38 e3. doi: 10.1016/j.celrep.2018.03.050 29617662 PMC5916809

[3] Loffler MW Chandran PA Laske K Schroeder C Bonzheim I Walzer M. Personalized peptide vaccine-induced immune response associated with long-term survival of a metastatic cholangiocarcinoma patient. J Hepatol (2016) 65(4):849–55. doi: 10.1016/j.jhep.2016.06.027 PMC5756536 27397612

[4] Ott PA Hu Z Keskin DB Shukla SA Sun J Bozym DJ. An immunogenic personal neoantigen vaccine for patients with melanoma. Nature (2017) 547(7662):217–21. doi: 10.1038/nature22991 PMC5577644 28678778

[5] Sahin U Derhovanessian E Miller M Kloke BP Simon P Lower M. Personalized rna mutanome vaccines mobilize poly-specific therapeutic immunity against cancer. Nature (2017) 547(7662):222–6. doi: 10.1038/nature23003 28678784

[6] Wick W Dietrich PY Kuttruff S Hilf N Frenzel K Admon A. Gapvac-101: first-in-human trial of a highly personalized peptide vaccination approach for patients with newly diagnosed glioblastoma. J Clin Oncol (2018) 36(15):2000–. doi: 10.1200/JCO.2018.36.15_suppl.2000

[7] Nelde A Rammensee HG Walz JS. The peptide vaccine of the future. Mol Cell Proteomics (2021) 20:100022. doi: 10.1074/mcp.R120.002309 33583769 PMC7950068

[8] Kote S Pirog A Bedran G Alfaro J Dapic I. Mass spectrometry-based identification of MHC-associated peptides. Cancers (Basel) (2020) 12(3):535. doi: 10.3390/cancers12030535 32110973 PMC7139412

[9] Becker JP Riemer AB. The importance of being presented: target validation by immunopeptidomics for epitope-specific immunotherapies. Front Immunol (2022) 13:883989. doi: 10.3389/fimmu.2022.883989 35464395 PMC9018990

[10] Nelde A Kowalewski DJ Stevanovic S. Purification and identification of naturally presented MHC class I and Ii ligands. Methods Mol Biol (2019) 1988:123–36. doi: 10.1007/978-1-4939-9450-2_10 31147937

[11] Bassani-Sternberg M. Mass spectrometry based immunopeptidomics for the discovery of cancer neoantigens. Methods Mol Biol (2018) 1719:209–21. doi: 10.1007/978-1-4939-7537-2_14 29476514

[12] Chong C Marino F Pak H Racle J Daniel RT Muller M. High-throughput and sensitive immunopeptidomics platform reveals profound interferongamma-mediated remodeling of the human leukocyte antigen (HLA) ligandome. Mol Cell Proteomics (2018) 17(3):533–48. doi: 10.1074/mcp.TIR117.000383 PMC5836376 29242379

[13] Eng JK Jahan TA Hoopmann MR. Comet: an open-source Ms/Ms sequence database search tool. Proteomics (2013) 13(1):22–4. doi: 10.1002/pmic.201200439 23148064

[14] Eng JK McCormack AL Yates JR. An approach to correlate tandem mass spectral data of peptides with amino acid sequences in a protein database. J Am Soc Mass Spectrom (1994) 5(11):976–89. doi: 10.1016/1044-0305(94)80016-2 24226387

[15] Zhang J Xin L Shan B Chen W Xie M Yuen D. Peaks Db: de novo sequencing assisted database search for sensitive and accurate peptide identification. Mol Cell Proteomics (2012) 11(4):M111 010587. doi: 10.1074/mcp.M111.010587 PMC3322562 22186715

[16] Falk K Rötzscheke O Stevanović S Jung G Rammensee HG. Allele-specific motifs revealed by sequencing of self-peptides eluted from MHC molecules. Nature (1991) 351:290–6. doi: 10.1038/351290a0 1709722

[17] Fritsche J Kowalewski DJ Backert L Gwinner F Dorner S Priemer M. Pitfalls in HLA ligandomics-how to catch a Li(E)Gand. Mol Cell Proteomics (2021) 20:100110. doi: 10.1016/j.mcpro.2021.100110 34129939 PMC8313844

[18] Verheggen K Raeder H Berven FS Martens L Barsnes H Vaudel M. Anatomy and evolution of database search engines-a central component of mass spectrometry based proteomic workflows. Mass Spectrom Rev (2020) 39(3):292–306. doi: 10.1002/mas.21543 28902424

[19] Klatt MG Mack KN Bai Y Aretz ZEH Nathan LI Mun SS. Solving an MHC allele-specific bias in the reported immunopeptidome. JCI Insight (2020) 5(19):e141264 . doi: 10.1172/jci.insight.141264 32897882 PMC7566711

[20] Sturm T Sautter B Worner TP Stevanovic S Rammensee HG Planz O. Mild acid elution and MHC immunoaffinity chromatography reveal similar albeit not identical profiles of the HLA class I immunopeptidome. J Proteome Res (2021) 20(1):289–304. doi: 10.1021/acs.jproteome.0c00386 33141586 PMC7786382

[21] Bernhardt M Cruz-Garcia Y Rech A Meierjohann S Erhard F Schilling B. Extending the mass spectrometry-detectable landscape of MHC peptides by use of restricted access material. Anal Chem (2022) 94(41):14214–22. doi: 10.1021/acs.analchem.2c02198 36194871

[22] Nicastri A Liao H Muller J Purcell AW Ternette N. The choice of HLA-associated peptide enrichment and purification strategy affects peptide yields and creates a bias in detected sequence repertoire. Proteomics (2020) 20(12):e1900401. doi: 10.1002/pmic.201900401 32359108

[23] Kall L Canterbury JD Weston J Noble WS MacCoss MJ. Semi-supervised learning for peptide identification from shotgun proteomics datasets. Nat Methods (2007) 4(11):923–5. doi: 10.1038/nmeth1113 17952086

[24] Schuler MM Nastke MD Stevanovic S. Syfpeithi: database for searching and T-cell epitope prediction. Methods Mol Biol (2007) 409:75–93. doi: 10.1007/978-1-60327-118-9_5 18449993

[25] Reynisson B Alvarez B Paul S Peters B Nielsen M. NetMHCpan-4.1 and netMHCiipan-4.0: improved predictions of MHC antigen presentation by concurrent motif deconvolution and integration of Ms MHC eluted ligand data. Nucleic Acids Res (2020) 48(W1):W449–W54. doi: 10.1093/nar/gkaa379 PMC7319546 32406916

[26] Reynisson B Barra C Kaabinejadian S Hildebrand WH Peters B Nielsen M. Improved prediction of MHC Ii antigen presentation through integration and motif deconvolution of mass spectrometry MHC eluted ligand data. J Proteome Res (2020) 19(6):2304–15. doi: 10.1021/acs.jproteome.9b00874 32308001

[27] Heberle H Meirelles GV da Silva FR Telles GP Minghim R. Interactivenn: A web-based tool for the analysis of sets through venn diagrams. BMC Bioinf (2015) 16:169. doi: 10.1186/s12859-015-0611-3 PMC4455604 25994840

[28] Kyte J Doolittle RF. A simple method for displaying the hydropathic character of a protein. J Mol Biol (1982) 157:150–32. doi: 10.1016/0022-2836(82)90515-0 7108955

[29] Kowalewski DJ Schuster H Backert L Berlin C Kahn S Kanz L. HLA ligandome analysis identifies the underlying specificities of spontaneous antileukemia immune responses in chronic lymphocytic leukemia (Cll). Proc Natl Acad Sci U.S.A. (2015) 112(2):E166–75. doi: 10.1073/pnas.1416389112 PMC4299203 25548167

[30] Backert L Kowalewski DJ Walz S Schuster H Berlin C Neidert MC. A meta-analysis of HLA peptidome composition in different hematological entities_ entity-specific dividing lines and “Panleukemia” Antigens. Oncotarget (2017) 8(27):43915–24. doi: 10.18632/oncotarget.14918 PMC5546449 28159928

[31] Nelde A Maringer Y Bilich T Salih HR Roerden M Heitmann JS. Immunopeptidomics-guided warehouse design for peptide-based immunotherapy in chronic lymphocytic leukemia. Front Immunol (2021) 12:705974. doi: 10.3389/fimmu.2021.705974 34305947 PMC8297687

[32] Klatt MG Kowalewski DJ Schuster H Di Marco M Hennenlotter J Stenzl A. Carcinogenesis of renal cell carcinoma reflected in HLA ligands: A novel approach for synergistic peptide vaccination design. Oncoimmunology (2016) 5(8):e1204504. doi: 10.1080/2162402X.2016.1204504 27622074 PMC5007970

[33] Reustle A Di Marco M Meyerhoff C Nelde A Walz JS Winter S. Integrative -omics and HLA-ligandomics analysis to identify novel drug targets for ccrcc immunotherapy. Genome Med (2020) 12(1):32. doi: 10.1186/s13073-020-00731-8 32228647 PMC7106651

[34] Walter S Weinschenk T Stenzl A Zdrojowy R Pluzanska A Szczylik C. Multipeptide immune response to cancer vaccine ima901 after single-dose cyclophosphamide associates with longer patient survival. Nat Med (2012) 18(8):1254–61. doi: 10.1038/nm.2883 22842478

[35] Vita R Mahajan S Overton JA Dhanda SK Martini S Cantrell JR. The immune epitope database (Iedb): 2018 update. Nucleic Acids Res (2019) 47(D1):D339–D43. doi: 10.1093/nar/gky1006 PMC6324067 30357391

[36] Perez-Riverol Y Bai J Bandla C Garcia-Seisdedos D Hewapathirana S Kamatchinathan S. The pride database resources in 2022: A hub for mass spectrometry-based proteomics evidences. Nucleic Acids Res (2022) 50(D1):D543–D52. doi: 10.1093/nar/gkab1038 PMC8728295 34723319

[37] Sheikh QM Gatherer D Reche PA Flower DR. Towards the knowledge-based design of universal influenza epitope ensemble vaccines. Bioinformatics (2016) 32(21):3233–9. doi: 10.1093/bioinformatics/btw399 PMC5079473 27402904

[38] Khodadoust MS Olsson N Wagar LE Haabeth OA Chen B Swaminathan K. Antigen presentation profiling reveals recognition of lymphoma immunoglobulin neoantigens. Nature (2017) 543(7647):723–7. doi: 10.1038/nature21433 PMC5808925 28329770

[39] Purcell AW Ramarathinam SH Ternette N. Mass spectrometry-based identification of MHC-bound peptides for immunopeptidomics. Nat Protoc (2019) 14(6):1687–707. doi: 10.1038/s41596-019-0133-y 31092913

[40] Klaeger S Apffel A Clauser KR Sarkizova S Oliveira G Rachimi S. Optimized liquid and gas phase fractionation increases HLA-peptidome coverage for primary cell and tissue samples. Mol Cell Proteomics (2021) 20:100133. doi: 10.1016/j.mcpro.2021.100133 34391888 PMC8724927

[41] Hoenisch Gravel N Nelde A Bauer J Mühlenbruch L Schroeder S Neidert MC. Timstof mass spectrometry-based immunopeptidomics refines tumor antigen identification. Research Square [Preprint] (2023). Available at: https://www.researchsquare.com/article/rs-2402111/v1 (Accessed 27.04.2023). 10.1038/s41467-023-42692-7 PMC10656517 37978195

[42] Pandey K Mifsud NA Lim Kam Sian TCC Ayala R Ternette N Ramarathinam SH. In-depth mining of the immunopeptidome of an acute myeloid leukemia cell line using complementary ligand enrichment and data acquisition strategies. Mol Immunol (2020) 123:7–17. doi: 10.1016/j.molimm.2020.04.008 32387766

[43] Chowell D Krishna S Becker PD Cocita C Shu J Tan X. Tcr contact residue hydrophobicity is a hallmark of immunogenic cd8+ T cell epitopes. Proc Natl Acad Sci U.S.A. (2015) 112(14):E1754–62. doi: 10.1073/pnas.1500973112 PMC4394253 25831525

[44] Calis JJ Maybeno M Greenbaum JA Weiskopf D De Silva AD Sette A. Properties of MHC class I presented peptides that enhance immunogenicity. PloS Comput Biol (2013) 9(10):e1003266. doi: 10.1371/journal.pcbi.1003266 24204222 PMC3808449

