# Supplementary material for: Immunoprecipitation methods impact the peptide repertoire in immunopeptidomics
Source: Front Immunol. 2023 Jul 21;14:1219720. doi: 10.3389/fimmu.2023.1219720 (PMC10400765; doi:10.3389/fimmu.2023.1219720)
Supplement: Supplementary file 1 [file DataSheet_1.docx]

Supplementary Material

Immunoprecipitation methods impact the peptide repertoire in immunopeptidomics

Marcel Wacker^1,2,3†^, Jens Bauer^1,2,3†^, Laura Wessling^2^, Marissa Dubbelaar^1,2,3,4^, Annika Nelde^1,2,3^, Hans‑Georg Rammensee^2,3,5^ and Juliane S. Walz^1,2,3,5,6*^

1 Department of Peptide-based Immunotherapy, University and University Hospital Tübingen, Tübingen, Germany

2 Institute for Cell Biology, Department of Immunology, University of Tübingen, Tübingen, Germany

3 Cluster of Excellence iFIT (EXC2180) “Image-Guided and Functionally Instructed Tumor Therapies”, University of Tübingen, Tübingen, Germany

4 Quantitative Biology Center (QBiC), University of Tübingen, Tübingen, Germany

5 German Cancer Consortium (DKTK) and German Cancer Research Center (DKFZ), partner site Tübingen, Tübingen, Germany

6 Clinical Collaboration Unit Translational Immunology, German Cancer Consortium (DKTK), Department of Internal Medicine, University Hospital Tübingen, Tübingen, Germany

† These authors contributed equally to this work and share the first authorship

* Correspondence:

Prof. Dr. med. Juliane Sarah Walz

Otfried-Müller-Str. 10, 72076 Tübingen, Germany

Phone: 0049-7071-2987305

Fax: 0049-7071-2925108

Mail: [juliane.walz@med.uni-tuebingen.de](mailto:juliane.walz@med.uni-tuebingen.de)

## Supplementary Table 1: Sample overview.

Sample overview with information on HLA typing, the used cell numbers/weights for each preparation, and the preparation method.

| Sample | HLA-Typing | Replicate Number | cell number/weight | origin | condition |
| --- | --- | --- | --- | --- | --- |
| JY | A*02:01; B*07:02; C*07:02; DRB1*04:04;DRB1*13:01; DQA1*01:03;DQA1*03:01;  DQB1*03:02;DQB1*06:03; DPA1*01:03; DPB1*02:01;DPB1*04:01 | JY1 | 1x10^8^ | Split from one pellet with 6x10^8^ cells | column |
|  |  | JY2 | 1x10^8^ |  |  |
|  |  | JY3 | 1x10^8^ |  |  |
|  |  | JY4 | 1x10^8^ |  |  |
|  |  | JY5 | 1x10^8^ |  |  |
|  |  | JY6 | 1x10^8^ |  |  |
|  |  | JY7 | 1x10^8^ | Split from one pellet with 6x10^8^ cells | 96-well |
|  |  | JY8 | 1x10^8^ |  |  |
|  |  | JY9 | 1x10^8^ |  |  |
|  |  | JY10 | 1x10^8^ |  |  |
|  |  | JY11 | 1x10^8^ |  |  |
|  |  | JY12 | 1x10^8^ |  |  |
|  |  | JY13 | 1x10^8^ | Split from one pellet with 6x10^8^ cells | column |
|  |  | JY14 | 1x10^8^ |  |  |
|  |  | JY15 | 1x10^8^ |  |  |
|  |  | JY16 | 1x10^8^ | Split from one pellet with 6x10^8^ cells | 96-well |
|  |  | JY17 | 1x10^8^ |  |  |
|  |  | JY18 | 1x10^8^ |  |  |
| CLL | A*02:01;A*11:01; B*35:01;B*40:01; C*04:01;C*03:04; DRB1*13:02:01;DRB1*15:01:01; DQA1*01:02:01; DQB1*06:02:01;DQB1*06:04:01; DPA1*01:03:01; DPB1*03:01:01;DPB1*04:01:01G | CLL1 | 3x10^8^ | Split from one pellet with 2.6x10^9^ cells | column |
|  |  | CLL2 | 3x10^8^ |  |  |
|  |  | CLL3 | 3x10^8^ |  |  |
|  |  | CLL4 | 3x10^8^ | Split from one pellet with 2.6x10^9^ cells | column |
|  |  | CLL5 | 3x10^8^ |  |  |
|  |  | CLL6 | 3x10^8^ |  |  |
|  |  | CLL7 | 3x10^8^ |  |  |
|  |  | CLL8 | 3x10^8^ |  |  |
|  |  | CLL9 | 3x10^8^ |  |  |
|  |  | CLL10 | 3x10^8^ | Split from one pellet with 2.6x10^9^ cells | 96-well |
|  |  | CLL11 | 3x10^8^ |  |  |
|  |  | CLL12 | 3x10^8^ |  |  |
|  |  | CLL13 | 3x10^8^ | Split from one pellet with 2.6x10^9^ cells | 96-well |
|  |  | CLL14 | 3x10^8^ |  |  |
|  |  | CLL15 | 3x10^8^ |  |  |
|  |  | CLL16 | 3x10^8^ |  |  |
|  |  | CLL17 | 3x10^8^ |  |  |
|  |  | CLL18 | 3x10^8^ |  |  |
| RCC | A*11:01;A*24:02; B*07:02;B*51:01; C*15:02;C*07:02; DRB1*09:01:02;DRB1*15:01:01; DQA1*01:02:01;DQA1*03:02:01; DQB1*03:03:02;DQB1*06:02:01;  DPA1*01:03:01;DPB1*04:01:01G | RCC1 | 0.83 g | Split from 2.8 g tissue | column |
|  |  | RCC2 | 0.83 g |  |  |
|  |  | RCC3 | 0.83 g |  |  |
|  |  | RCC4 | 0.83 g | Split from 2.5 g tissue | column |
|  |  | RCC5 | 0.83 g |  |  |
|  |  | RCC6 | 0.83 g |  |  |
|  |  | RCC7 | 0.83 g | Split from 2.7 g tissue | column |
|  |  | RCC8 | 0.83 g |  |  |
|  |  | RCC9 | 0.83 g |  |  |
|  |  | RCC10 | 0.83 g | Split from 3.5 g tissue | 96-well |
|  |  | RCC11 | 0.83 g |  |  |
|  |  | RCC12 | 0.83 g |  |  |
|  |  | RCC13 | 0.83 g | Split from 2.7 g tissue | 96-well |
|  |  | RCC14 | 0.83 g |  |  |
|  |  | RCC15 | 0.83 g |  |  |
|  |  | RCC16 | 0.83 g | Split from 2.9 g tissue | 96-well |
|  |  | RCC17 | 0.83 g |  |  |
|  |  | RCC18 | 0.83 g |  |  |

## Supplementary Table 2: List of identified HLA class I ligands.

List of all identified peptide sequences for the column preparation method, 96-well method, desalting step, or 50% ACN elution steps. In addition to the peptide sequence, further information on the cell type, replication number, preparation method, GRAVY score, retention time, area, q-value, XCorr value, and best NetMHCpan 4.1 or SYFPEITHI binder is given.

## Supplementary Table 3: List of identified HLA class II ligands.

List of all identified peptide sequences for the column preparation method, 96-well method, desalting step, or 50% ACN elution steps. In addition to the peptide sequence, further information on the cell type, replication number, preparation method, GRAVY score, retention time, area, q-value, and XCorr value, best NetMHCIIpan 4.1 rank and allele and the core sequence are given.

## Supplementary Table 4: List of identified method-exclusive peptides.

List of all method-exclusive peptide sequences for the column preparation method, 96-well method, desalting step, or 50% ACN. The different sheets depict exclusive peptides of the comparisons column vs. 96-well, column vs. desalting, 96-well vs. 50% ACN and column vs. the combination of 96-well and 50% ACN. In addition to the peptide sequence, further information on the cell type, replication number, preparation method, GRAVY score, retention time, accession number, q-value, and XCorr value are given.

## Supplementary Table 5: List of peptides used for the comparison with published tumor-associated antigens

List of published tumor-associated antigens used for the comparison with the here identified peptides. In addition to the peptide sequence, the T cell response and reference are given. It is also indicated how often the respective peptide was found by which immunoprecipitation method.

## Supplementary Table 6: Immune Epitope Database (IEDB) peptide list

IEDB peptide lists which were used for the overlap analysis with column-based and 96-well-based immunopeptidome peptide lists. In addition to the peptide sequence, the peptide length is also given. Furthermore, the filter settings and information on the further filtering process are stored in a separate sheet.

## Supplementary Table 7: Immunogenicity scores of 9-mer peptides.

Lists of peptide sequences of 9-mers with their corresponding immunogenicity scores.


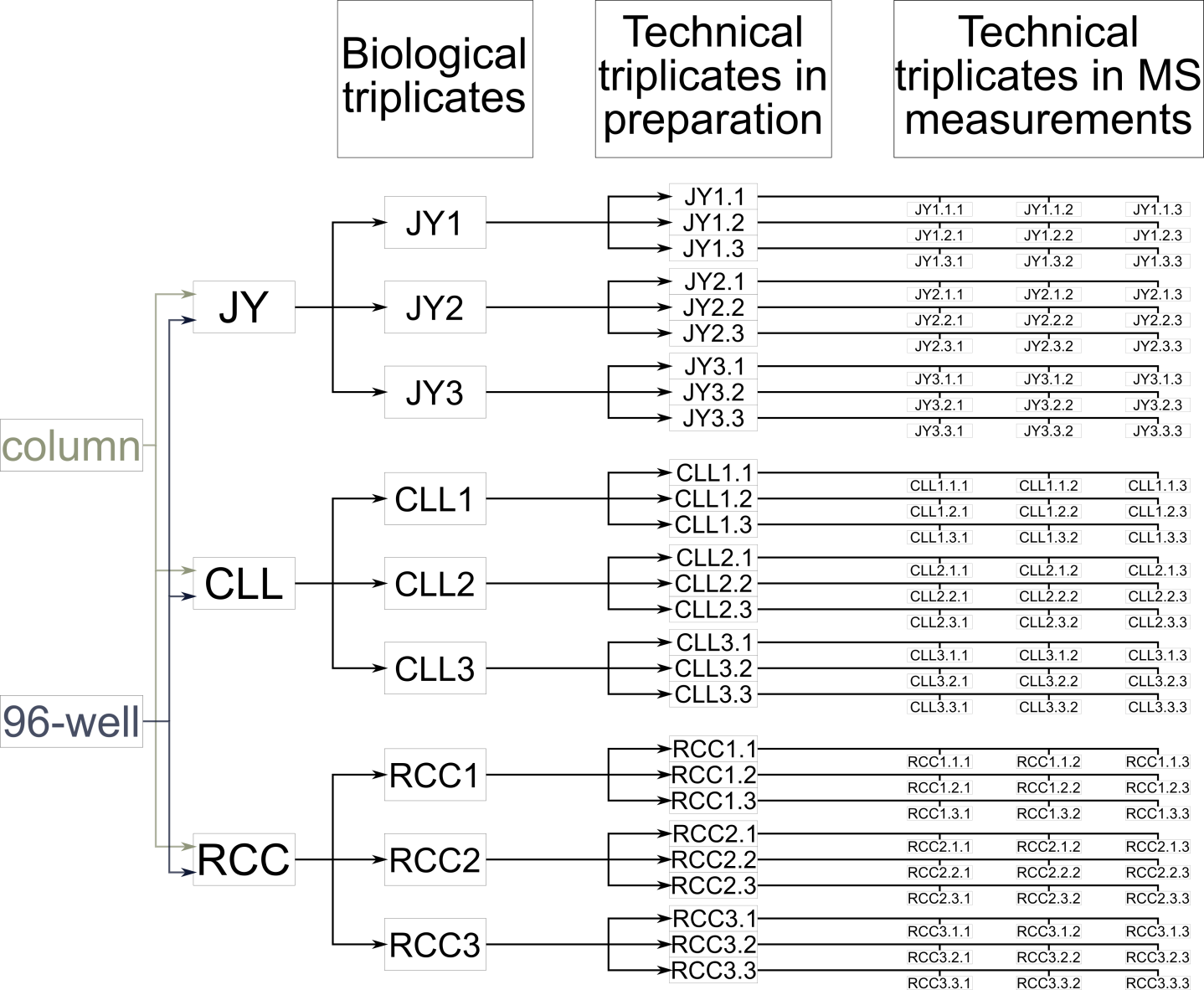


## Supplementary Figure 1: Schematic representation of the sample overview.

Three different sample types were used: cells of the JY cell line, peripheral blood mononuclear cells (PBMCs) of a chronic lymphocytic leukemia (CLL) patient, and a solid tumor tissue sample of a renal cell carcinoma (RCC) patient. Three biological replicates were used for each sample type (the material was divided equally). For each biological replicate, three technical replicates in terms of separate immunoprecipitations were performed per method. Subsequent MS measurements were also performed in three technical replicates. Thus, 27 measurements for HLA class I and HLA class II are available for each sample type and method, respectively. Technical MS replicates were co-processed, producing 9 samples for JY, CLL, and RCC for each method and HLA class.


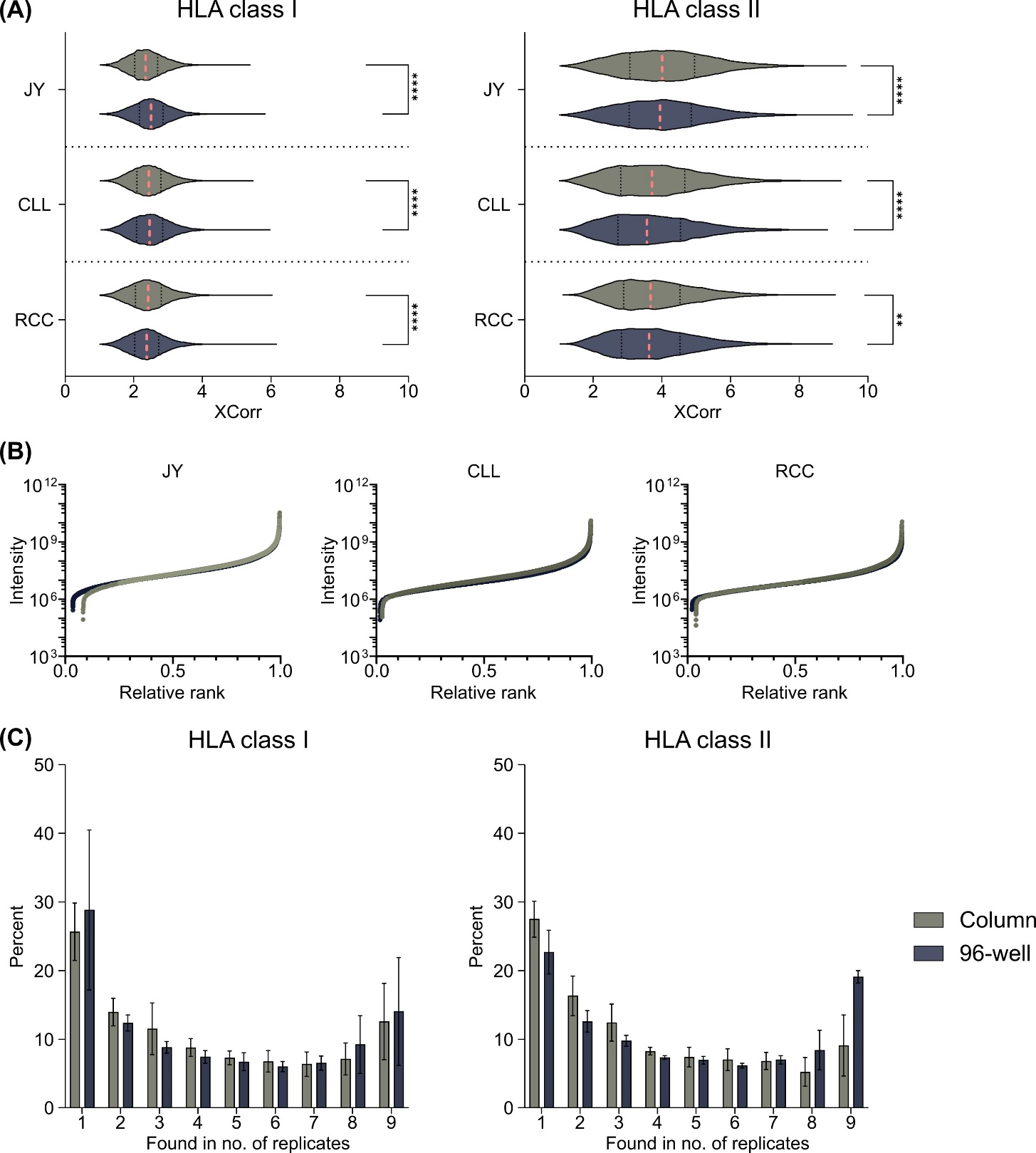


## Supplementary Figure 2: Quality and reproducibility analysis of HLA restricted peptides isolated with the 96-well and column HLA preparation methods.

**(A)** Violin plots of calculated XCorr values from all identified peptides. **(B)** Relative ranked intensities of MS-acquired data derived from all samples' combined HLA class II immunopeptidomes (n = 9). **(C)** Relative overlap analysis of peptide occurrences in replicates depicted as bar diagrams. For each specimen, the relative peptide occurrence was calculated and the results were combined in this graph. Red dashed lines show the median, black dotted lines the 25^th^ and 75^th^ percentiles. ****p<0.0001, **p<0.01.


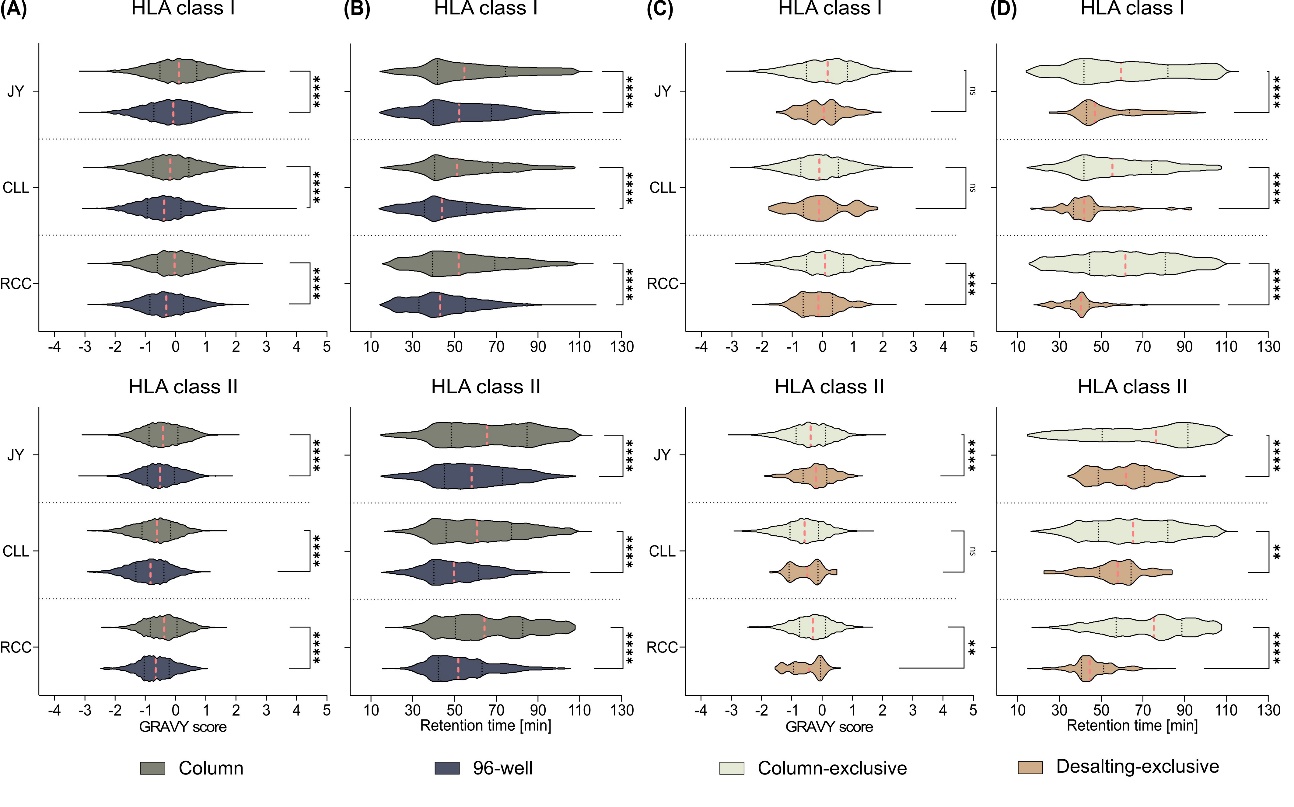


## Supplementary Figure 3: Hydrophobicity scores and retention times of all column or 96-well peptides and the role of desalting-associated peptides.

**(A)** Violin plots of the grand average of hydropathy (GRAVY) scores and **(B)** retention times violin plots of all peptides found in the column or 96-well immunopeptidomes of HLA class I (upper panel) and HLA class II (lower panel) peptides. **(C)** Violin plots of the GRAVY scores and **(D)** violin plots of the retention times of HLA class I (upper panel) or HLA class II (lower panel) exclusive peptides either found with the column preparation method or in the desalting solution. Red dashed lines show the median, black dotted lines the 25^th^ and 75^th^ percentiles. **p<0.01, ***p<0.001, ****p<0.0001, ns not significant. **(A** and **C)** unpaired t-tests**, (B** and **D)** Mann-Whitney tests. Peptides found with the column method are shown in green, peptides found with the 96-well method in dark gray-blue, and peptides unique to the desalting step in light brown.


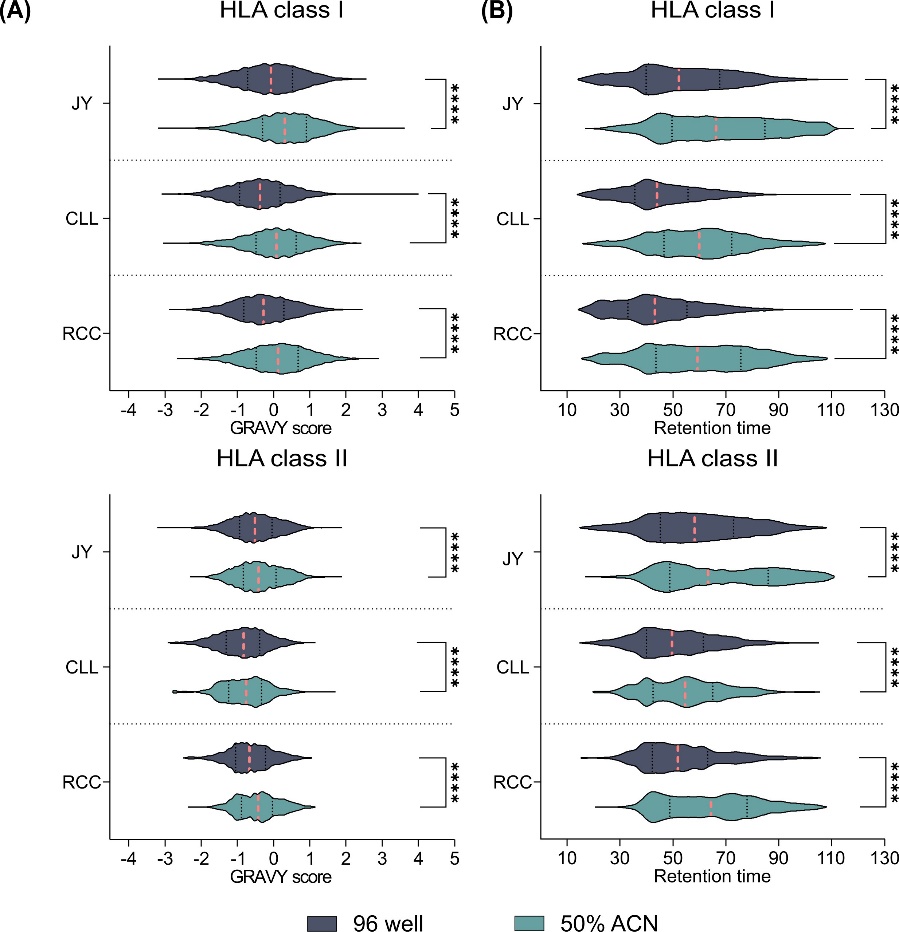


## Supplementary Figure 4: Hydrophobicity scores and retention times of all 96-well or 50% ACN peptides.

**(A)** Violin plots GRAVY scores and **(B)** violin plots of the retention times of all HLA class I (upper panel) or HLA class II (lower panel) peptides found in the 96-well or 50% ACN immunopeptidomes. Red dashed lines show the median, black dotted lines the 25^th^ and 75^th^ percentiles. ****p<0.0001, **(A)** unpaired t-tests**, (B)** Mann-Whitney tests. Peptides found with the 96-well method are in dark gray-blue, and peptides found with 50% ACN are in blue-green.


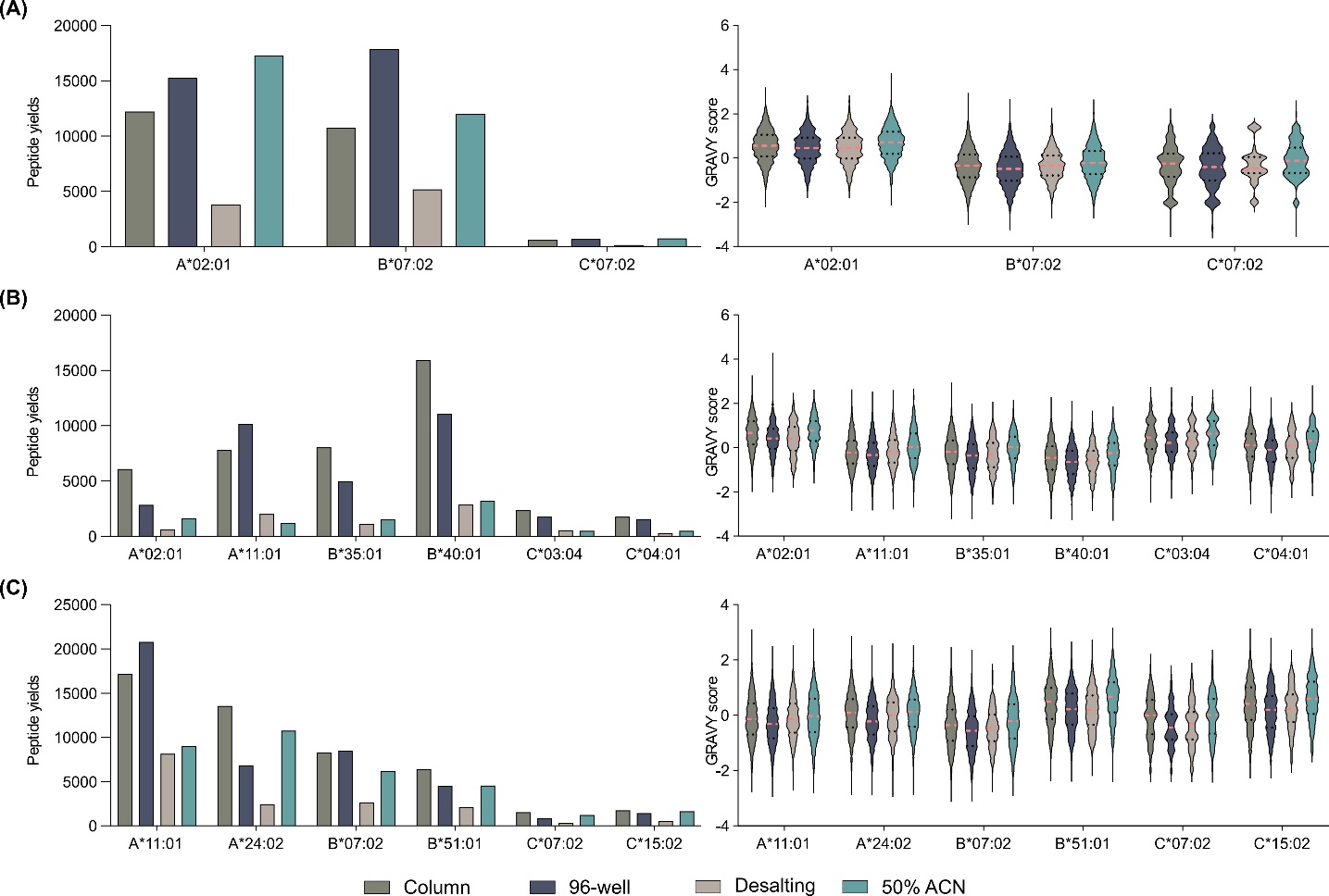


## Supplementary Figure 5: Allotype-specific peptide yields and hydrophobicity scores of the examined methods and method adaptions

Bar diagram (left panel) and violin plots (right panel) of allotype-specific peptide yields and hydrophobicity scores of **(A)** JY, **(B)** CLL, and **(C)** RCC. Red dashed lines show the median, black dotted lines the 25^th^ and 75^th^ percentiles. Dark green depicts all column-based peptides; gray-blue depicts all 96-well peptides; light brown depicts all desalting peptides; 50% of ACN peptides are in blue-green.
